# *Mycobacterium abscessus*: Environmental Bacterium Turned Clinical Nightmare

**DOI:** 10.3390/microorganisms7030090

**Published:** 2019-03-22

**Authors:** Rose C. Lopeman, James Harrison, Maya Desai, Jonathan A. G. Cox

**Affiliations:** 1 School of Life and Health Sciences, Aston University, Aston Triangle, Birmingham B4 7ET, UK; lopemarc@aston.ac.uk (R.C.L.); j.harrison11@aston.ac.uk (J.H.); 2 Birmingham Children’s Hospital, Birmingham Women’s and Children’s NHS Foundation Trust, Steelhouse Lane, Birmingham B4 6NH, UK; maya.desai@nhs.net

**Keywords:** *Mycobacterium abscessus*, non-tuberculous mycobacteria, antimicrobial drug discovery, cystic fibrosis

## Abstract

Mycobacteria are a large family of over 100 species, most of which do not cause diseases in humans. The majority of the mycobacterial species are referred to as nontuberculous mycobacteria (NTM), meaning they are not the causative agent of tuberculous (TB) or leprosy, i.e., *Mycobacterium tuberculous* complex and *Mycobacterium leprae*, respectively. The latter group is undoubtedly the most infamous, with TB infecting an estimated 10 million people and causing over 1.2 million deaths in 2017 alone TB and leprosy also differ from NTM in that they are only transmitted from person to person and have no environmental reservoir, whereas NTM infections are commonly acquired from the environment. It took until the 1950′s for NTM to be recognised as a potential lung pathogen in people with underlying pulmonary disease and another three decades for NTM to be widely regarded by the medical community when *Mycobacterium avium* complex was identified as the most common group of opportunistic pathogens in AIDS patients. This review focuses on an emerging NTM called *Mycobacterium abscessus* (*M. abs*). *M. abs* is a rapidly growing NTM that is responsible for opportunistic pulmonary infections in patients with structural lung disorders such as cystic fibrosis and bronchiectasis, as well as a wide range of skin and soft tissue infections in humans. In this review, we discuss how we came to understand the pathogen, how it is currently treated and examine drug resistance mechanisms and novel treatments currently in development. We highlight the urgent need for new and effective treatments for *M. abs* infection as well as improved in vivo methods of efficacy testing.

## 1. Introduction

*Mycobacterium abs* was first isolated in 1952 by Moore and Frerichs from a 63-year-old woman’s knee abscess [1] and since then, our understanding of the pathogen has rapidly and somewhat turbulently expanded. When it was first isolated, it was suggested by the authors that *M. abs* was an entirely new species of nontuberculous mycobacteria (NTM) and was given its name due to its ability to produce subcutaneous abscesses. Interestingly, at this point, *M. abs* was considered to be a pathogen of low virulence due to the perception that it was primarily a pathogen causing cutaneous infections that appeared transient and self-limiting [1]. 40 years after its discovery, *M. abs* was first implicated in pulmonary infections after an analysis of 154 patients with rapidly growing mycobacteria (RGM) pulmonary infections revealed that 82% of the isolates were *M. abs*; the disease was considered to be slowly progressive but virulent nonetheless [2]. This was preceded by an observation of four patients with pulmonary disease caused by the related organism *Mycobacterium chelonae*; however, the cause of these infections cannot be confirmed as *M. abs* [3]. Since its first identification, *M. abs* nomenclature and species/subspecies identification have undergone many changes.

In 1952 [1], *M. abs* was believed to be identical to *M. chelonae*, another RGM that infects fish and amphibians, as it presented identical biochemical features [4]. Then, in 1972, following an international collaborative study by the International Working Group on Mycobacterial Taxonomy, *M. abs* was designated subspecies status [4]. 20 years later, in 1992, Kusunoki and Ezaki used DNA hybridisation to establish that there is only 35% DNA relatedness between *M. chelonae* subsp. *chelonae* and *M. chelonae* subsp. *abscessus*. In light of this, *M. abs* was finally re-elevated to species status [5].

However, in 2004, an unusual *mycobacterium* was isolated from a patient with hemoptoic pneumonia, and researchers were unable to accurately identify the species using the techniques described above. They developed partial polymerase chain reaction (PCR) sequencing of the *rpoB* gene and were able to demonstrate that the isolate shared 96.0% partial *rpoB* sequence similarity and a 98.0% *recA* gene sequence similarly with only the *M. abs* type strain. They had previously proposed that *rpoB* gene sequence difference of >3% and a *recA* gene sequence difference of >2% was sufficient to differentiate between different NTM species. Using this new *rpoB* gene sequencing technique aided with the more traditional biochemical assays and 16S rRNA gene sequencing, the authors were able to produce an accurate phylogenetic tree of various NTM. They concluded that this novel isolate was a new species closely related to and likely recently derived from *M. abs*. This was subsequently named *Mycobacterium massiliense* [6].

In 2006, *rpoB* gene sequencing was used on 59 clinical isolates of RGM [7], and they found that 15.3% of these isolates were novel, corresponding to three new species of mycobacteria. One of these species, named *Mycobacterium bolletii* by the authors, was found to share 100% 16S gene similarity and 95.6% *rpoB* gene sequence similarity with *M. abs*.

Over the years, many different biochemical and molecular techniques have been employed to identify NTM species. Up until the early 2000s, the sodium chloride tolerance test was used to identify species of RGM, particularly in distinguishing between *M. abs* and *M. chelonae* species, as *M. abs* is able to grow on Löwenstein-Jensen medium with 5% sodium chloride, but *M. chelonae* is not [8]. However, several investigators reported that this method is unreliable, likely due to vague criteria and the cross-over of biochemical features between differing species of RGM [8,9,10]. The citrate utilization assay perhaps provides more reliability, the premise being that *M. abs* is unable to use citrate as a carbon source whereas other RGM such as *M. chelonae* are [11]. As is also the case with the sodium chloride test, this assay takes up to 8 weeks to complete and therefore is losing traction in the clinical setting [11]. High Performance Liquid Chromatography (HPLC) has also been used to generate mycolic acid patterns and thus distinguish between RGM species; however, this technique has limitations as several RGM have similar mycolic acid profiles [12]. Despite its widespread use in species identification, 16S rRNA sequencing has been shown to be inadequate for species identification of mycobacteria [6]. An assay with superior specificity was needed to differentiate between NTM species and subspecies.

**Figure 1:**
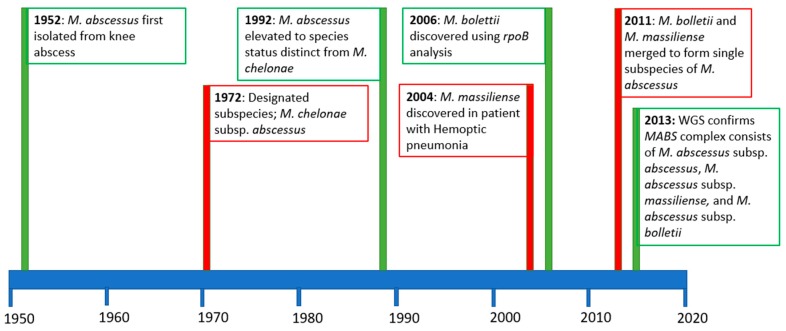
Timeline of *Mycobacterium abscessus* taxonomy from 1950 through to the present day. In the first 50 years since its discovery, no congruent terminology was in widespread use to accurately describe and differentiate *M. abs* from other nontuberculous mycobacteria (NTM). In the mid-2000s, improved molecular technology resulted in the discovery of the two *M. abscessus* subspecies; *M. abscessus* subsp. *massiliense* and *M. abscessus* subsp. *bolletii* in 2004 and 2006, respectively. Then, in 2011, it was proposed that *M. abscessus* subsp. *massiliense* and *M. abscessus* subsp. *bolletii* should be merged into one subspecies, *M. abscessus* subsp. *massiliense*. This caused some confusion within the medical community, until in 2013, when whole genome sequencing (WGS) showed genetic divisions that clearly identified the three subspecies within the *M. abs* complex.

In 2011 it was proposed by Leao et al. [13] that the *M. abscessus* complex (*MABS* complex) should be amended to include *M. abscessus* subsp. *abscessus* (as before) and to combine the two subspecies to form one single subspecies, *M. abscessus* subsp. *bolletii.* Finally, in 2013, whole-genome sequencing (WGS) was used by Bryant et al. to identify transmission between patients with cystic fibrosis (CF) [14]. The authors subjected 168 clinical isolates of *M. abs* to WGS and a phylogenetic tree produced from the isolates showed clearly, for the first time, that *M. abscessus* subsp. *abscessus, M. abscessus* subsp. *bolletii*, and *M. abscessus* subsp. *massiliense* are three distinct subspecies belonging to the *MABS* complex. The idea that *MABS* is a complex that contains three subspecies that are genetically very similar, but phenotypically divergent was given more traction in 2016 when Tortoli et al., [15] published an amended description of the *MABS* complex that highlighted the importance of subspecies differentiation. The authors argued that the criteria for subspecies as proposed by Wayne et al., [16] i.e., “genetically close organisms that diverge in phenotype” is appropriate in this case, considering the genetic similarity and the presence of an inducible and functional *erm*(41) gene conferring macrolide resistance in only *M. abscessus* subsp. *bolletii* and *M. abscessus* subsp. *abscessus* isolates whereas *M. abscessus* subsp. *massiliense* has a non-functional *erm*(41) gene. Trovato et al. identified that WGS was effective at discriminating *M. abscessus* subsp. *abscessus* from other isolates with more accuracy than *rpoB* gene sequencing [17]. An important recent study using WGS on 32 *M. abs* isolates, identified multiple subpopulations within each patient, demonstrating that isolates from sputum do not represent the entire *M. abs* diversity within a patient. This has serious implications for isolate sensitivity testing and subsequent infection management [18]. The timeline of *M. abs* taxonomy is summarised in Figure 1. It is clear that WGS of clinical isolates is of vital importance for effective diagnosis and targeted treatment for *M. abs* infection.

## 2. *M. abscessus* and Cystic Fibrosis

NTM species are ubiquitous in the environment (unlike *M. tuberculosis* and *M. leprae*, which require a living host and are transmitted patient to patient or zoonotically), suggesting that NTM exposure is extremely common, whereas NTM disease is still relatively rare. Those with pre-existing lung diseases undoubtedly have some predisposition to NTM infection, leading some to describe a “two-hit” theory of NTM disease acquisition [19]. Undoubtedly, the leading population affected by *M. abs* is the CF population. However, there have also been incidences of *M. abs* infections in non-CF populations.

CF is an autosomal recessive disorder caused by mutations in the CF transmembrane conductance regulator gene (CFTR). Despite being a multi-organ disease, one of the most prominent features in CF is chronic pulmonary infection. The major pathogen associated with lung infection in CF is *Pseudomonas aeruginosa*, and unfortunately, 80 to 90% of patients with CF die from respiratory failure as a result of chronic bacterial infection [20]. Even from infancy, the lungs of CF patients are already commonly colonised with a variety of organisms such as *Staphylococcus aureus* and *Haemophilus influenzae*. Before 1990, NTM infection was not often associated with CF. However, since then, reports of *M. abs* infection (along with other NTM species) have been increasingly common. Several large-scale studies have been performed over the past decade or so, revealing an NTM prevalence in CF patients in some areas as high as 20% (Table 1).

Age is a strong correlator of NTM infection in this group, with 40% of CF patients over the age of 40 having NTM smear positive results, as opposed to 4–20% in the under-40s population [26]. Other risk factors for NTM infection in CF patients appears to be lower body mass index (BMI) values, worse forced expiratory volume (FEV_1_), current infection with *Pseudomonas aeruginosa* and *Stenotrophomonas maltophilia*, experience of pneumothorax requiring chest drain, the use of inhaled antibiotics and other medical interventions. [27]. Recently, a longitudinal study identified that NTM infection is increasing in prevalence in the UK pediatric CF population from 1.3% to 3.8% between 2010 and 2015 with a sample size of 5333 patients under 16 years of age [28]. One study performed in Israel found a significant association between *Aspergillus* species and NTM species in sputum cultures of CF patients [29].

*M. abs* also causes serious disseminated infections following transplantation [30]. A single case study involving post-transplant *M. abs* skin and soft tissue infection (SSTI) resulted in disseminated pulmonary infection and eventually the death of the patient, despite aggressive pre- and peri-operative anti-mycobacterial therapy [31]. For this reason, many have recommended that *M. abs* colonisation should be viewed as a contraindication to lung transplantation. This suggestion, however, has been met with criticism. Some studies have shown that it is possible to perform a lung transplant on patients with *M. abs* colonisation and that subsequent clearance of infection is possible, albeit with a strong possibility of severe complications [32,33]. Despite this uncertainty surrounding the outcome of lung transplantation in patients colonised with *M. abs*, it is increasingly clear that effective treatments for *M. abs* lung infection must be developed, as lung transplantation is a potentially life-saving therapy for end-stage lung disease caused by CF and other lung disorders.

## 3. *M. abscessus* Infection in Non-CF Populations

*M. abs* infection can also occur in non-CF populations. It is well documented that a risk factor for NTM pulmonary disease is patients with low body fat. The mechanisms behind this are not well understood; however, it is possible that leptin plays a role in NTM predisposition [34].

Aside from pulmonary infections, *M. abs* is also able to produce SSTIs in otherwise healthy hosts. There have been cases of *M. abs* outbreaks following the use of contaminated needles and other surgical instruments [35] and even, as was the case in a cohort of ‘lipotourists’ (i.e., people who travel abroad for cosmetic surgery for fat removal), severe outbreaks following cosmetic surgery [36]. Interestingly, *M. abs* has also been linked to late-onset wound infections following crush trauma sustained by Swedish survivors of the 2004 tsunami that killed over 200,000 people and caused serious crush injuries in another >2000 [37]. *M. abs* pulmonary infections in non-CF patients have been previously reviewed [38].

## 4. Environmental Reservoirs and Transmission

NTM are ubiquitous in the environment; especially water sources and soil [1]. They are prone to biofilm formation and this contributes to their ability to persist in harsh environments [39]. NTM can persist in environments that are in close proximity to human populations, particularly human water sources, hospital water supplies (sinks, showerheads) and homes.

*M. abs*, like other NTM, is able to survive in harsh, nutrient-starved environments where other competing microorganisms would not survive, such as in chlorinated water [40]. The presence of the lipid-rich cell wall results in a hydrophobic cell surface, which facilitates the formation of biofilms, their slow growth and adherence to surfaces, thus aiding their survival and providing them with a selective advantage [41,42,43]. Furthermore, many RGM are oligotrophic, requiring low levels of two carbon sources and minimal amounts of metal ions [44], further indicating their hardiness and persistence in harsh environments. The impenetrable nature of the *M. abs* cell wall in comparison to other non-mycobacterial pathogens also contributes to its resistance to many antibiotics and disinfectants [45,46]. The ability of *M. abs* to survive in the human environment presents a huge problem for human health, with most studies up until this point suggesting that patients with CF predominately acquire NTM infection from the environment [14]. This long-held belief was called into question in 2013 when Floto and his team used WGS to show possible patient to patient transmission of *M. abs* within a CF clinic in the UK [47].

In 2009, Feazel et al. demonstrated that showerheads provide an enriched environment for NTM biofilm formation; the presence of human pathogens including NTM were >100 fold higher in showerhead biofilms compared to the background water contents [48]. A study in Hawaii investigated the prevalence of NTM in household plumbing; areas such as showerheads, sinks, taps, shower drains and refrigerator water dispensers were sampled. The authors found that 69% of households surveyed had clinically significant NTM colonisation, of which 10% was *M. abs* [49]. Another 2018 study revealed an outbreak of *M. abs* skin infections in children who were exposed to the same indoor wading pool [50]. This study demonstrates the importance of identifying *M. abs* environmental reservoirs, reporting *M. abs* cases and subsequent environmental remediation in order to reduce the risk of infection.

The persistence and spread of NTM species within healthcare environments is fast becoming a serious problem and a significant threat to human health [51]. It was a long-held belief in the scientific community that NTM is transmitted to humans from the environment, and that patient to patient transmission is unlikely. This resulted in a clinical focus on reducing the risk of environmental transmission using effective sterilising techniques and other hygiene practices. Such as it is, the CF Trust published *M. abs* infection control recommendations that include general infection control measures such as hand washing and more specific recommendations such as segregation of infected patients from other patients [52].

The mode of transmission of pathogenic NTM to humans is still poorly understood, with many studies seeking evidence of human to human transmission using molecular techniques such as WGS. A study undertaken in 2001 sought to address this question; a retrospective analysis of 1062 respiratory specimens taken from 214 patients with CF revealed five patients with *M. abs* lung infection. These five patients each had isolates with a unique genotype that was not shared with any of the other patients, which led the authors to conclude that patient to patient transmission of *M. abs* was not occurring within their cohort [53].

In 2014, a small-scale study was performed on 27 *M. abs* isolates from 20 paediatric CF patients [54]. The authors used a combination of epidemiology, variable number tandem repeat (VNTR) profiling and WGS to find evidence of cross-infection between paediatric CF patients. It was hypothesized that patients with strains that had identical VNTR profiles would have had intense exposure to each other compared with patients with strains that had different VNTR profiles. Little evidence of transmission between patients was found, except for two patients who were siblings and therefore had higher intensity of exposure. The authors concluded that cross-infection was uncommon in their cohort, and that transmission is most likely to be from a common environmental source [54].

The biggest shift in our understanding of transmission came in 2013 when a major study was published in which WGS was used to identify transmission of *M. abs* between patients at an adult CF centre in the UK between 2007 and 2011 [14]. The authors found a high level of relatedness between isolates of *M. abscessus* subsp. *abscessus*, but clusters were clearly segregated from one another, indicating that patients have independently acquired either genetically diverse strains or a dominant circulating clone. However, in the case of *M. abscessus* subsp. *massiliense* the authors found isolates from different individuals with almost identical genomic sequences, strongly indicating transmission between patients. Analysis of the environment revealed no NTM species isolated from the water supply to the clinic, showerheads, dish washers, bronchoscopes or the local River Cam or Papworth Hospital Pond. Further investigation into possible transmission routes revealed patients with isolates from the same genetic relatedness clusters were present in the clinic at the same time as each other, further supporting their hypothesis that *M. abscessus* subsp. *massiliense* is likely transmitted from patient to patient rather than independently from the environment. This finding represents a major clinical advance which may require patients infected with *M. abs* to be segregated from *M. abs*-naïve patients to prevent onward transmission.

Following on from the localised retrospective study published in 2013 [14], a global WGS initiative was launched on 1080 isolates from 517 patients from the UK, USA, Republic of Ireland, mainland Europe and Australia [47]. This study found that the majority of isolates were from densely clustered genotypes that were not diverse, suggesting a high level of human-human transmission. Phylogenetic analysis also revealed that there are three dominant circulating clones globally, and these clones are associated with higher virulence and poor clinical outcomes. Human-human transmission appears to have facilitated the evolution of *M. abs* from an environmental pathogen to a transmissible human pathogen.

## 5. Diagnosis and Treatment:

As *M. abs* and other NTM species are ubiquitous in the environment, including drinking water supplies, the presence of culture-positive respiratory tract sample for NTM does not always indicate NTM-pulmonary disease (NTM-PD). Therefore, patients must also have characteristic symptoms, compatible radiology, and two or more positive sputum samples for the same NTM species, as well as the exclusion of other potential causes of pulmonary disease [55].

For clinical laboratory identification of NTM species, the British Thoracic Society (BTS) recommends that isolates be obtained from sputum samples, and if this is not possible (for example in children), bronchoalveolar lavage or transbronchial biopsy samples should be taken when NTM pulmonary disease is suspected [55]. NTM infection can be validated in the laboratory, with the use of auramine-phenol staining and microscopy, as well as culture on solid and liquid media.

All clinical isolates of *M. abs* undergo susceptibility testing for clarithromycin, cefoxitin and amikacin. They also recommend that other antibiotics such as tigecycline, imipenem, minocycline, moxifloxacin and clofazimine are tested in this manner [55].

## 6. Treatment

When *M. abs* was first isolated in 1952, it was thought the patient was initially infected with the pathogen at the age of 14 years old. The patient’s condition resolved without intervention and so for some time, treatment wasn’t considered a priority in *M. abs* infections [1].

Of course, today it is well known that treatment for *M. abs* pulmonary infection is essential to give the patient the best chance of survival. Unfortunately, antimicrobial chemotherapy for *M. abs* infection is particularly difficult due to its intrinsic and acquired resistance to most of the commonly used antibiotic classes. Further complications in the treatment of *M. abs* infection is the lack of evidence that in vitro susceptibility of antibiotics corresponds to in vivo efficacy in treating pulmonary disease [26]. Because chemotherapy-based treatment of *M. abs* infection is often unsuccessful, the American Thoracic Society advises that certain patients may have the best chance of disease regression with resectional surgery, especially if the patient exhibits a poor response to drug therapy, if macrolide-resistance develops, or if the patient is experiencing disease-related complications such as haemoptysis [26].

**Figure 2:**
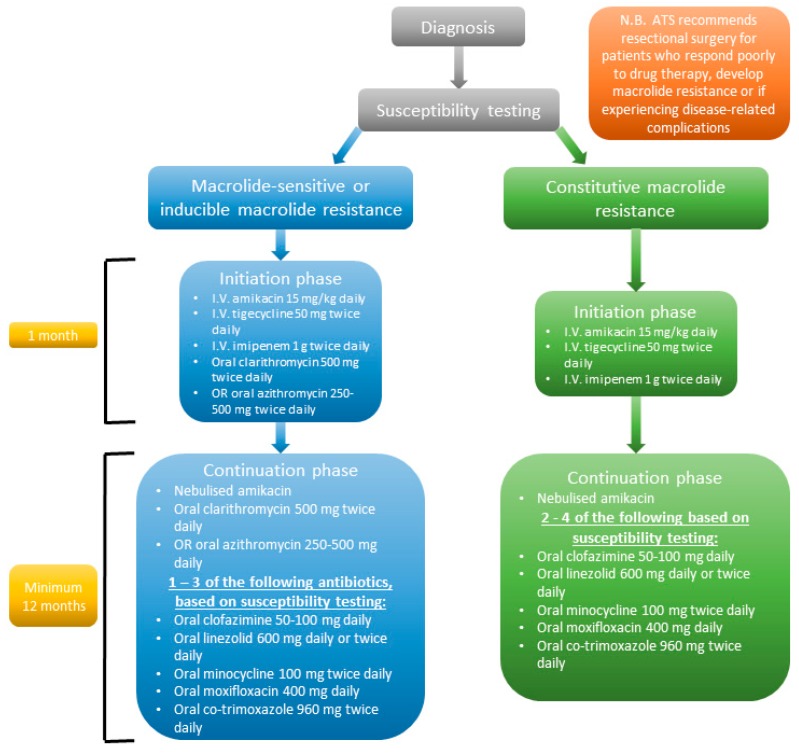
Flow chart showing treatment regimen for *M. abs*-pulmonary disease based on laboratory susceptibility testing results as recommended by the British Thoracic Society. Treatment will differ based on the whether the isolate displays macrolide sensitivity/inducible macrolide resistance or constitutive macrolide resistance. The initial phase of treatment involves three intravenous (I.V.) antibiotics, and for macrolide sensitive/inducible macrolide resistance one of two oral macrolides, and this phase lasts one month. The continuation phase also depends on laboratory susceptibility testing results and clinicians will typically administer 1-4 oral antibiotics over a period of at least 12 months. It is also important to note that the American Thoracic Society (ATS) recommends surgical resection of infected area if the patient is not responding to therapy, if macrolide resistance develops and/or if the patient develops disease-related complications such as haemoptysis.

Current treatment guidelines from the BTS [55] recommend that treatment for *M. abs* pulmonary disease should consist of an initial phase antibiotic regimen that includes intravenous (I.V.) and oral antibiotics, followed by a continuation phase comprising of oral and inhaled antibiotics (Figure 2). Further genetic analysis of clinical isolates can provide information on the *erm*(41) (inducible macrolide resistance) and/or presence of 23S rRNA point mutation (constitutive macrolide resistance) in clinical isolates of *M. abs*, which can then be used to inform patient-specific treatment regimens.

Side effects of *M. abs* treatment are common and can be severe. A retrospective analysis of 65 patients undergoing treatment for *M. abs* lung disease in South Korea [56] revealed frequent adverse reactions to cefoxitin; 51% of patients developed leukopenia, 6% of patients developed thrombocytopenia, and 15% of patients experienced drug-induced hepatotoxicity. As a result, cefoxitin was discontinued in 60% of patients and side effects resolved. Another common side effect observed was gastrointestinal problems (nausea, anorexia, or diarrhoea), which affected 22% of patients and caused four patients (6%) to completely stop antibiotic treatment. A clinical recommendation was made to consider imipenem as an alternative to cefoxitin; however, prolonged treatment with imipenem can cause neutropenia.

Another study that analysed treatment outcomes in 65 patients with *M. abs* in North America also found a high prevalence of side effects. IV amikacin (65% of patients) and azithromycin (71% of patients) were the most commonly used antimicrobials in this cohort. They found 74 different side effects reported in 62% of patients, most commonly nausea/vomiting (31%) and skin changes (20%). They attributed many of these side effects to amikacin or tigecycline, and as a result, of those received amikacin or tigecycline therapy, 51% and 36% of patients, respectively, had to adjust or stop medication due to severe side effects such as ototoxicity. Similar to the South Korean study, four patients had to totally stop treatment because of their side effects [57].

Clarithromycin is one of the most commonly-used antibiotics to treat *M. abs* [26]. However, clarithromycin has been associated with hearing loss, with one study citing a 7% hearing loss rate in their patients. This side effect did resolve in all but one patient; however, the authors state that the patient had a pre-existing condition that hindered their ability to attribute this hearing loss solely to clarithromycin [58]. A case study on an 81-year-old woman, who was being treated with clarithromycin for infective exacerbation of chronic pulmonary obstructive disease (COPD) showed another example of clarithromycin-related permanent hearing loss, despite evidence that clarithromycin is relatively well tolerated [59,60]. The major issue with using clarithromycin to treat *M. abs* is the presence of a functional inducible *erm*(41) gene that confers macrolide resistance in both *M. abscessus* subsp. *abscessus* and *M. abscessus* subsp. *bolletii* but not *M. abscessus* subsp. *massiliense*.

### The Resistance Problem: Why the Drugs Don’t Work

*M. abs* is known for its intrinsic resistance to most chemotherapeutic agents, including all the anti-tuberculous drugs used to treat *M. tuberculosis* infection [35,61]. Furthermore, in vitro drug susceptibility testing on *M. abs* often proves unhelpful in guiding treatment regimens [62]. There are a number of natural resistance mechanisms displayed by *M. abs* (along with other mycobacteria), including a waxy and impermeable cell wall, drug export systems, antibiotic modifying/inactivating enzymes and genetic polymorphism of target genes [45].

The greatest contributing factor to the lack of *M. abs* sensitivity to many major classes of antibiotic is the mycobacterial cell wall, the role of which has long been studied. The high lipid content and unusual thickness of the mycobacterial cell wall provides an effective barrier for hydrophilic and lipophilic agents [63]. In 1990 it was shown that the lack of permeability of the *M. chelonae* (then grouped together with *M. abs*) cell wall plays a vital role in making the pathogen resistant to antibiotics [46]. The cell wall barrier is also responsible for *M. abs*’ intrinsic resistance to acids and alkalis [64]. The cell wall of mycobacteria also contains porins; it was shown in 1990 that *M. chelonae* possesses a 59 kDa cell wall protein that allows for the diffusion of small, hydrophilic solutes. However, this porin is minor, unlike that of *E. coli* where they are the most abundant cell wall protein, explaining the low permeability to hydrophilic solutes [65]. The cell wall cannot explain all of the intrinsic drug resistance seen in *M. abs*; in fact, it is known that the cell wall, particularly the porins, act synergistically with internal systems that are activated by the presence of intracellular antibiotics, and that the low permeability of the mycobacterial cell wall means that the bacteria has time to induce the expression of drug resistance genes [65].

As a constituent of the mycobacterial cell wall, active efflux pumps can be described as one of the main causative factors of drug resistance in mycobacteria [45,66,67]. They primarily act to protect bacteria against toxic compounds and promote bacterial homeostasis by transporting toxins or metabolites to the extracellular environment [67]. *M. abs* encodes protein members of the major facilitator family ATP-binding cassette (ABC) transporters as well as mycobacterial membrane protein large (MmpL) families [68]. ABC transporters are found in all forms of life and make use of adenosine triphosphate (ATP) to transport molecules across membranes. The MmpL transporter family is a subclass of a large family of multidrug resistance pumps known as Resistance-Nodulation-Cell-Division (RNCD) permeases. MmpLs export lipid components across the cell envelope of mycobacteria [69]. The role of MmpLs in *M. abs* drug resistance is yet to be fully understood; however, there is evidence that MmpL7 in *M. tuberculosis* confers resistance to isoniazid [70], suggesting that MmpLs may play a major role.

Macrolides are one of the mainstays of *M. abs* treatment [26], yet despite this, *M. abs* infections tend to respond poorly to macrolide therapy, even when they appear sensitive to clarithromycin in vitro [71]. A study performed in 2009 revealed the presence of an inducible *erm*(41) gene in 7 out of 10 *M. abs* clinical isolates that confers resistance to macrolides with a minimum inhibitory concentration (MIC) of ≥32 µg/mL. The three remaining susceptible isolates had *erm*(41) gene; however, it appeared to be non-functional [71]. The *erm*(41) gene produces a functional 23S rRNA methylase, contributing to macrolide resistance along with point mutations in the *rrl* encoding 23S rRNA gene [72]. Following on from this, it was shown that macrolides may be useful in treating approximately 20% of *M. abs* infections in the U.S., and that sequencing of the *erm*(41) gene is a potentially useful tool in predicting macrolide susceptibility [73]. It is also noteworthy that *M. abscessus* subsp. *massiliense* contains a large 97 base pair deletion in *erm*(41), rendering it useless. Therefore, *M. abscessus* subsp. *massiliense* retains susceptibility to macrolides, except in the case of *rrl* mutants [71,74,75,76]. *M. abs* isolates possessing an *rrl* mutant display constitutive resistance to macrolide antibiotics. This phenomenon is known to be mediated by a mutation in *rrl* encoding the bacterial 23S rRNA gene, particularly at positions 2058 and 2059, i.e., the drug binding pocket of the gene [77].

If macrolide therapy is not advised due to evidence of constitutive resistance, there are of course other chemotherapeutic options available. However, in many of the conserved genes in *M. abs* that can potentially act as drug targets, there is the presence of genetic polymorphisms, which can often confer drug resistance [45].

A 1998 study revealed an amino acid substitution at position 83 (Ser83Ala) in the quinolone-resistance-determining-region (QRDR) in fluoroquinolone-resistant isolates of *M. abs* [78]. This substitution occurs in the region of DNA gyrase subunit *GyrA* that binds DNA, and as fluoroquinolones bind strongly to the gyrase-DNA complex, and weakly to protein or DNA alone, this mutation results in fluoroquinolone resistance [79]. Genetic polymorphisms also occur within the *emb* operon that codes for several homologous arabinosyl transferases. These are enzymes involved in the polymerisation of arabinogalactan, an essential component of the mycobacterial cell wall and can be inhibited by the tuberculosis drug ethambutol. A 1997 study showed that polymorphisms at position 306 in a highly conserved *embB* gene conferred natural resistance across many species of mycobacteria, including *M. abs* [79]. *M. abs* has high natural levels of resistance to ethambutol (MIC >64mg/L), and the same study transferred the *M. abs emb* region to ethambutol-susceptible *M. smegmatis* resulted in a 500-fold increase in the MIC to ethambutol [61].

*M. abs* also produces a number of target-modifying enzymes. Rifampicin ADP-ribosyl transferase, Arr_*Mab* inactivates rifamycins such as rifampicin. Aminoglycoside 2′-N-acetyltransferase and aminoglycoside phosphotransferases mediate the susceptibility to aminoglycoside antibiotics. *M. abs* has also been shown to produce an endogenous β-lactamase (Bla_Mab_), which efficiently hydrolyses the β-lactam ring of β-lactam antibiotics, rendering them ineffective [61,80,81].

Aside from antibiotic-specific internal drug resistance mechanisms, a family of transcriptional regulators, the WhiB family, is excusive to actinomycetes and may be involved in conferring drug resistance in *M. abs*. Members of this family have been shown to regulate systems of drug resistance in *M. tuberculosis*, including antibiotic export and activation [82]. *M. abs* has been shown to possess a homologue of the *M. tuberculosis WhiB7*. When *M. abs WhiB7* is deleted, the result is increased sensitivity to clinically relevant antibiotics that target the ribosome, such as clarithromycin, amikacin and tetracycline [83].

## 7. Future Perspectives for *M. abscessus*

### Future Treatments

It perhaps goes without saying that there is an urgent, unmet need for safe and effective treatments against *M. abs* pulmonary disease. There have been instances of successful treatment of *M. abs* with already available antibiotics. One such case was reported in 2002, where a 63-year-old patient whose infection had not responded to the traditional regimen was prescribed a course of faropenem, a new member of the β-lactam antibiotic class. Treatment was successful and produced no adverse side effects [84]. It is not just antimicrobials that have potential in enhancing *M. abs* treatment. In 2012, Okazaki et al. reported that the use of clarithromycin, amikacin and imipenem/cilastatin to treat a case of *M. abs* pulmonary was greatly enhanced with the addition of corticosteroids. The authors recommend that the presence of organising pneumonia (a non-specific inflammatory pulmonary process) or an allergic reaction may have helped to explain the poor response to antibiotic treatment alone in some patients, and that this possibility should be considered when applicable to improve treatment outcomes [85].

**Figure 3:**
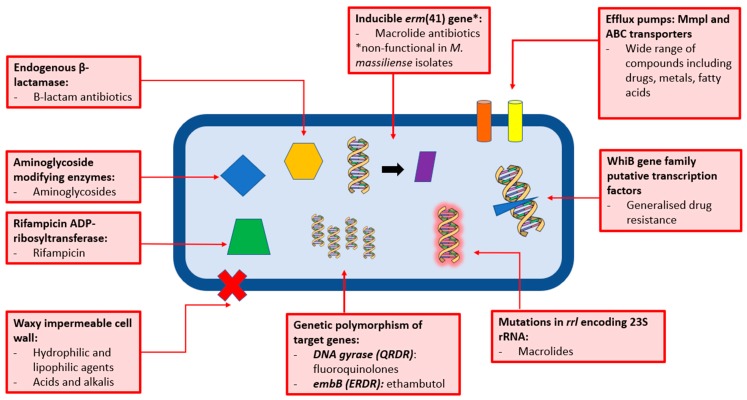
Graphical summary of the resistance mechanisms exhibited by *Mycobacterium abscessus* (*M. abs*). There are several mechanisms involving different physiological, enzymatic and genomic processes that contribute to the notoriously drug-resistant profile of *M. abs*. It is likely that these processes, such as efflux pumps and drug resistance genes, work in synergy to produce a highly resistant pathogen.

One of the enzymatic resistance mechanisms employed by *M. abs* is the production of an endogenous β-lactamase, Bla_Mab_ (Figure 3). Cefoxitin and imipenem, both β-lactam antibiotics, are commonly used to treat *M. abs*. In order to improve the efficacy of these antibiotics, a β-lactamase inhibitor may be administered in conjunction during therapeutic treatment. A 2015 study revealed that avibactam, a β-lactamase inhibitor is able to efficiently inhibit Bla_Mab_ [86], and a subsequent 2017 study showed that avibactam improves the efficacy of imipenem against *M. abs* both in vitro and in macrophage and zebrafish models of infection [87].

Aside from these examples, very few case studies have reported successful treatment with repurposed antibiotics. Therefore, novel drug targets in *M. abs* must be discovered and elucidated, and novel compounds that safely and effectively inhibit these targets discovered.

**Figure 4:**
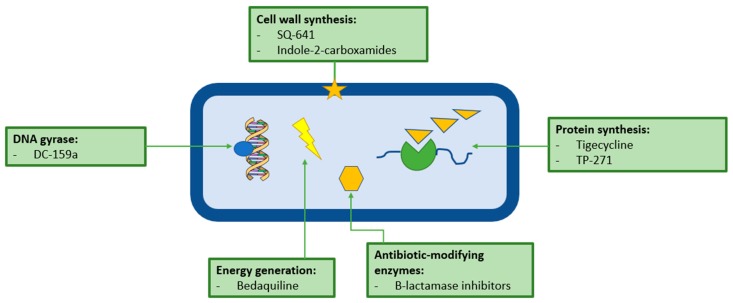
Graphical summary of the exploitable drug targets in *Mycobacterium abscessus* (*M. abs*). There are several potential target areas in *M. abs* including physiological, genomic, enzymatic and metabolic processes. Many of the drugs with potential to be used as part of *M. abs* treatment are old classes of antibiotics that have been repurposed, such as β-lactamase inhibitors, or have been discovered as part of the anti-tuberculous drug discovery pipelines, such as bedaquiline.

There are potentially a wide variety of viable drug targets in *M. abs* (Figure 4). Many of the most promising leads against *M. abs* have come about as a result of concerted effort to find novel drugs for *M. tuberculosis*, which a handful of researchers have applied to *M. abs* and other NTM species. Unfortunately, only a small percentage of the novel drugs which are active against *M. tuberculosis*, are also active against *M. abs*, further highlighting just how resistant and dangerous this pathogen is proving to be.

One potential target in *M. abs* is DNA gyrase, despite the fact that *M. abs* is naturally resistant to quinolones [78], a novel fluoroquinolone, DC-159a was developed in 2010 as part of the Working Group on TB Drugs, and was found to be active against *M. abs* with an MIC of 16 µg/mL, which was four to eight-fold lower than the other already available quinolones tested [88]. The authors stressed the importance of in vivo testing of DC-159a; however, no publications attesting to the in vivo activity of DC-159a against *M. abs* have been released to date.

The mycobacterial cell wall, in all its complexity, can offer an attractive range of potential antibiotic targets. The three distinct layers of the mycobacterial cell wall: core peptidoglycan, arabinogalactan and mycolic acids are each essential to the pathogen and involve a number of exploitable processes [89]. A 2010 study subjected several species of NTM to a capuramycin analogue SQ641 [90]. Capuramycins are a novel class of nucleoside antibiotics that work by targeting phosphor-*N*-acetylmuramyl-pentapeptide-translocase (translocase-1 or TL-1), which is essential for peptidoglycan synthesis. They found that the drug had an MIC of 0.25-1 µg/mL, as well as finding synergy between SQ641 and rifabutin and streptomycin. This drug has great potential as it is fast-acting and displays a long post-antibiotic effect [91]. In 2017 a study was published in which several members of the newly synthesized MmpL3 inhibitors, indole-2-carboxamides, have shown potent activity against *M. abs*. These inhibitors have been shown to work by inhibiting the transfer of mycolic acids to their cell envelope acceptors in *M. abs* strains [92]. Further work has been done on this class of inhibitors; in 2019, Pandya et al. reported that oral administration of the inhibitors shows a statistically significant reduction in bacterial load in the lungs and spleens of *M. abs*-infected mice [93].

It has been demonstrated that *M. abs* displays high levels of intrinsic resistance to the tetracycline class of antibiotics via the monooxygenase, MabTetX, a *WhiB7*-independent pathway [94]. This is not the end of the road for this class of antibiotics. Tigecycline, the first developed glycylcycline, a new class of tetracycline antibiotics originally developed for SSTIs, was shown in 2014 to be highly effective in vivo against *M. abs* pulmonary disease [95]. Further work in 2018 revealed that tigecycline is a poor substrate of MabTetX and is incapable of inducing its expression, explaining its high efficacy in comparison with other tetracycline antibiotics [94]. Tigecycline is now one of the recommended treatment options for *M. abs* pulmonary disease, and is arguably one of the most effective, with one study citing clinical improvement in >60% patients with *M. abs* pulmonary disease when tigecycline is employed as part of the multi-drug regimen against *M. abs* [95]. Tigecycline is not the only tetracycline showing activity against *M. abs*. A 2012 study tested the in vitro activity of a novel fluorocycline antibiotic, TP-271 (a tetracycline-related antibiotic) against 22 isolates of *M. abs*. They found all the isolates to have an MIC of ≤1 µg/mL with an average of 0.5 µg/mL, which is decidedly superior to that of the other orally available tetracycline antibiotics [96].

Bedaquilin, the latest drug indicated for the treatment of multi-drug resistant TB (MDR-TB) was approved by the FDA in 2011, and it works by targeting the ATP synthase of mycobacteria. Obregon et al. [97] demonstrated an MICs of 1.0 µg/mL against *M. abs* reference strain and then in 2017, Vesenbeckh et al. pointed to bedaquiline as a potential antimicrobial against *M. abs* after the drug exhibited MICs of ≤1 µg/mL against 20 *M. abs* clinical isolates in vivo [98]. In vitro activity has also been observed against a variety of *M. abs* isolates with both tedizolid and clofazimine; however, these compounds are yet to be tested for activity in vivo [99,100].

## 8. Summary

*M. abs* is increasingly being recognised as an important pathogen responsible for a wide range of infections and implicated in severe, and often untreatable pulmonary infections in people with CF and other structural lung disorders. Despite considerable recent progress, there remain many unanswered questions about this pathogen’s virulence, transmission and environmental persistence. Furthermore, almost all of the currently available antibiotics are useless against *M. abs*, with even official guideline treatment regimens having little to no evidence of in vivo efficacy. With such high treatment failure rates, clinicians are often forced to administer last-resort antibiotics in the hope of a cure. Coupled with increasing prevalence and its already extensively drug resistant profile, it is glaringly obvious that novel, effective and safe treatments are needed. Many of the novel drugs mentioned above are in various phases of clinical trial against *M. tuberculosis* and there is a significant paucity of data regarding their efficacy against *M. abs* and other NTM species. Furthermore, there is a startling lack of in vivo efficacy data for any of these drugs, which is particularly worrying considering the inconsistencies between in vitro and in vivo anti-*M. abs* activity. Whilst TB has many dedicated drug-discovery programmes, NTM has none. A dedicated NTM drug discovery pipeline is essential to ensure the disease burden of NTM does not become overwhelming.

## Figures and Tables

**Table 1:** Prevalence of non-tuberculous mycobacterial lung disease in cystic fibrosis patients in differing geographical areas between 2004 and 2014. CF: cystic fibrosis.

Study	Location	Sample Size	NTM Prevalence in CF
Oliver, KN (2004) [21]	USA	750	13% (majority *M. avium* complex)
Roux, AL, et al. (2009) [22]	France	1582	6.6% (*M. abs* most common)
Seddon, P, et al. (2013) [23]	UK	3805 adults 3317 children	5% adults 3.3% children
Adjemian, J, et al. (2014) [24]	USA	18,003	10–20%; depending on area
Mussaffi, H, et al. (2005) [25]	Israel	139	8.6%
